# Supplementary material for: Methodological and Practical Challenges in Synthesizing Occupational Cancer Studies
Source: Int J Environ Res Public Health. 2024 Jun 6;21(6):742. doi: 10.3390/ijerph21060742 (PMC11203818; doi:10.3390/ijerph21060742)
Supplement: Supplementary file 1 [file ijerph-21-00742-s001.zip › ijerph-2966822-supplementary.pdf]

Table S1. Studies used in Figure 1.

First Author	Publication Year	Incidence	Mortality	Enrollment	Place	State	Reference Number
Rosenstock	1990	0	1	1945-1980	Seattle	Washington	[1]
Rosenstock	1990	0	1	1945-1980	Tacoma	Washington	[1]
Rosenstock	1990	0	1	1945-1980	Portland	Oregon	[1]
Heyer	1990	0	1	1945-1983	Seattle	Washington	[2]
Lee	2020	1	0	1981-2014	Florida	Florida	[3]
Vena	1987	0	1	1950-1979	Buffalo	New York	[4]
Beaumont	1991	0	1	1940-1970	San Francisco	California	[5]
Demers	1992	0	1	1944-1979	Seattle	Washington	[6]
Demers	1992	0	1	1944-1979	Tacoma	Washington	[6]
Demers	1992	0	1	1944-1979	Portland	Oregon	[6]
Burnett	1994	0	1	1984-1990	Alaska	Alaska	[7]
Burnett	1994	0	1	1984-1990	Colorado	Colorado	[7]
Burnett	1994	0	1	1984-1990	Georgia	Georgia	[7]
Burnett	1994	0	1	1984-1990	Idaho	Idaho	[7]
Burnett	1994	0	1	1984-1990	Indiana	Indiana	[7]
Burnett	1994	0	1	1984-1990	Kansas	Kansas	[7]
Burnett	1994	0	1	1984-1990	Kentucky	Kentucky	[7]
Burnett	1994	0	1	1984-1990	Maine	Maine	[7]
Burnett	1994	0	1	1984-1990	Missouri	Missouri	[7]
Burnett	1994	0	1	1984-1990	Nebraska	Nebraska	[7]
Burnett	1994	0	1	1984-1990	Nevada	Nevada	[7]
Burnett	1994	0	1	1984-1990	New Hampshire	New Hampshire	[7]
Burnett	1994	0	1	1984-1990	New Jersey	New Jersey	[7]
Burnett	1994	0	1	1984-1990	New Mexico	New Mexico	[7]
Burnett	1994	0	1	1984-1990	New York	New York	[7]
Burnett	1994	0	1	1984-1990	North Carolina	North Carolina	[7]
Burnett	1994	0	1	1984-1990	Ohio	Ohio	[7]
Burnett	1994	0	1	1984-1990	Oklahoma	Oklahoma	[7]
Burnett	1994	0	1	1984-1990	Pennsylvania	Pennsylvania	[7]
Burnett	1994	0	1	1984-1990	Rhode Island	Rhode Island	[7]
Burnett	1994	0	1	1984-1990	South Carolina	South Carolina	[7]
Burnett	1994	0	1	1984-1990	Tennessee	Tennessee	[7]
Burnett	1994	0	1	1984-1990	Utah	Utah	[7]
Burnett	1994	0	1	1984-1990	Vermont	Vermont	[7]
Burnett	1994	0	1	1984-1990	Washington	Washington	[7]
Burnett	1994	0	1	1984-1990	West Virginia	West Virginia	[7]
Burnett	1994	0	1	1984-1990	Wisconsin	Wisconsin	[7]
Figgs	1995	1	0	1984-1989	Georgia	Georgia	[8]
Figgs	1995	1	0	1984-1989	Idaho	Idaho	[8]
Figgs	1995	1	0	1984-1989	Indiana	Indiana	[8]
Figgs	1995	1	0	1984-1989	Kansas	Kansas	[8]
Figgs	1995	1	0	1984-1989	Kentucky	Kentucky	[8]
Figgs	1995	1	0	1984-1989	Maine	Maine	[8]
Figgs	1995	1	0	1984-1989	Missouri	Missouri	[8]
Figgs	1995	1	0	1984-1989	Nebraska	Nebraska	[8]
Figgs	1995	1	0	1984-1989	Nevada	Nevada	[8]

Figgs	1995	1	0	1984-1989	New Hampshire	New Hampshire	[8]
Figgs	1995	1	0	1984-1989	New Jersey	New Jersey	[8]
Figgs	1995	1	0	1984-1989	New Mexico	New Mexico	[8]
Figgs	1995	1	0	1984-1989	North Carolina	North Carolina	[8]
Figgs	1995	1	0	1984-1989	Ohio	Ohio	[8]
Figgs	1995	1	0	1984-1989	Oklahoma	Oklahoma	[8]
Figgs	1995	1	0	1984-1989	Rhode Island	Rhode Island	[8]
Figgs	1995	1	0	1984-1989	South Carolina	South Carolina	[8]
Figgs	1995	1	0	1984-1989	Tennessee	Tennessee	[8]
Figgs	1995	1	0	1984-1989	Utah	Utah	[8]
Figgs	1995	1	0	1984-1989	Washington	Washington	[8]
Figgs	1995	1	0	1984-1989	West Virginia	West Virginia	[8]
Figgs	1995	1	0	1984-1989	Wisconsin	Wisconsin	[8]
Figgs	1995	1	0	1984-1989	Vermont	Vermont	[8]
Figgs	1995	1	0	1984-1989	Colorado	Colorado	[8]
Figgs	1995	1	0	1984-1989	Georgia	Georgia	[8]
Figgs	1995	1	0	1984-1989	Idaho	Idaho	[8]
Figgs	1995	1	0	1984-1989	Indiana	Indiana	[8]
Figgs	1995	1	0	1984-1989	Kansas	Kansas	[8]
Figgs	1995	1	0	1984-1989	Kentucky	Kentucky	[8]
Figgs	1995	1	0	1984-1989	Maine	Maine	[8]
Figgs	1995	1	0	1984-1989	Missouri	Missouri	[8]
Figgs	1995	1	0	1984-1989	Nebraska	Nebraska	[8]
Figgs	1995	1	0	1984-1989	Nevada	Nevada	[8]
Figgs	1995	1	0	1984-1989	New Hampshire	New Hampshire	[8]
Figgs	1995	1	0	1984-1989	New Jersey	New Jersey	[8]
Figgs	1995	1	0	1984-1989	New Mexico	New Mexico	[8]
Figgs	1995	1	0	1984-1989	North Carolina	North Carolina	[8]
Figgs	1995	1	0	1984-1989	Ohio	Ohio	[8]
Figgs	1995	1	0	1984-1989	Oklahoma	Oklahoma	[8]
Figgs	1995	1	0	1984-1989	Rhode Island	Rhode Island	[8]
Figgs	1995	1	0	1984-1989	South Carolina	South Carolina	[8]
Figgs	1995	1	0	1984-1989	Tennessee	Tennessee	[8]
Figgs	1995	1	0	1984-1989	Utah	Utah	[8]
Figgs	1995	1	0	1984-1989	Washington	Washington	[8]
Figgs	1995	1	0	1984-1989	West Virginia	West Virginia	[8]
Figgs	1995	1	0	1984-1989	Wisconsin	Wisconsin	[8]
Figgs	1995	1	0	1984-1989	Vermont	Vermont	[8]
Wende	1996	0	1	1950-1982	Buffalo	New York	[9]
Ma	1998	0	1	1984-1993	Florida	Florida	[10]
Krstev	1998	1	0	1986-1989	Atlanta	Georgia	[11]
Krstev	1998	1	0	1986-1989	Detroit	Michigan	[11]
Krstev	1998	1	0	1986-1989	New Jersey	New Jersey	[11]
Baris	2001	0	1	1925-1986	Philadelphia	Philadelphia	[12]
Krishnan	2003	1	0	1991-1994	San Francisco	California	[13]
Krishnan	2003	1	0	1997-1999	San Francisco	California	[13]
Ma	2005	0	1	1972-1999	Florida	Florida	[14]
Ma	2006	1	0	1981-1999	Florida	Florida	[15]
Bates	2007	1	0	1983-2003	California	California	[16]
Greene	2008	1	0	2006-2007	San Francisco	California	[17]

Zeig-Owens	2011	1	0	1996-2001	New York City	New York	[18]
Zeig-Owens	2015	1	0	1973-2011	New York City	New York	[19]
Karami	2012	1	0	2002-2007	Detroit	Michigan	[20]
Karami	2012	1	0	2002-2007	Chicago	Illinois	[20]
Daniels	2014	1	1	1950-2009	California	California	[21]
Daniels	2014	1	1	1950-2009	Chicago	Illinois	[21]
Daniels	2014	1	1	1950-2009	Philadelphia	Pennsylvania	[21]
Daniels	2014	1	1	1950-2009	California	California	[21]
Daniels	2014	1	1	1950-2009	Chicago	Illinois	[21]
Daniels	2014	1	1	1950-2009	Philadelphia	Pennsylvania	[21]
Daniels	2015	1	0	1950-2009	California	California	[22]
Daniels	2015	1	0	1950-2009	Chicago	Illinois	[22]
Daniels	2015	1	0	1950-2009	Philadelphia	Pennsylvania	[22]
Daniels	2015	1	0	1950-2009	California	California	[22]
Daniels	2015	1	0	1950-2009	Chicago	Illinois	[22]
Daniels	2015	1	0	1950-2009	Philadelphia	Pennsylvania	[22]
Tsai	2015	1	0	1988-2007	California	California	[23]
Moir	2016	1	0	2001-2009	New York City	New York	[24]
Muegge	2018	0	1	1985-2013	Indiana	Indiana	[25]
Landgren	2018	1	0	2011-2017	New York City	New York	[26]
Lenahan	2018	1	0	1979-2013	Newark	New Jersey	[27]
Lenahan	2018	1	0	1979-2013	Jersey City	New Jersey	[27]
Lenahan	2018	1	0	1979-2013	Paterson	New Jersey	[27]
Lenahan	2018	1	0	1979-2013	Elizabeth	New Jersey	[27]

Table S2. Studies used in Figure 2

First Author	Publication Year	Incidence	Mortality	Enrollments	Place	Country	Reference Number
Hansen	1990	0	1	1970-1980	Denmark	Denmark	[28]
Ide	1998	1	1	1985-1994	Strathclyde	UK	[29]
Ide	1998	1	1	1985-1994	Scotland	UK	[29]
Deschamps	1995	0	1	1977-1991	Paris	France	[30]
Tornling	1994	1	1	1931-1983	Stockholm	Stockholm	[31]
Kullberg	2018	1	0	1958-2012	Stockholm	Stockholm	[32]
Petersen	2018	1	0	1968-2014	Denmark	Denmark	[33]
Petersen	2018	0	1	1970-2014	Denmark	Denmark	[33]
Bigert	2016	1	0	1985-2009	Germany	Germany	[34]
Bigert	2016	1	0	1985-2009	Italy	Italy	[34]
Bigert	2016	1	0	1985-2009	France	France	[34]
Bigert	2016	1	0	1985-2009	Spain	Spain	[34]
Bigert	2016	1	0	1985-2009	Czech Republic	Czech Republic	[34]
Bigert	2016	1	0	1985-2009	Hungary	Hungary	[34]
Bigert	2016	1	0	1985-2009	Poland	Poland	[34]
Bigert	2016	1	0	1985-2009	Slovakia	Slovakia	[34]
Bigert	2016	1	0	1985-2009	Romania	Romania	[34]
Bigert	2016	1	0	1985-2009	Russia	Russia	[34]
Bigert	2016	1	0	1985-2009	UK	UK	[34]
Bigert	2016	1	0	1985-2009	Sweden	Sweden	[34]
Amadeo	2015	0	1	1979-2008	France	France	[35]
Pukkala	2014	1	0	1961-2005	Denmark	Denmark	[36]
Pukkala	2014	1	0	1961-2005	Finland	Finland	[36]
Pukkala	2014	1	0	1961-2005	Iceland	Iceland	[36]
Pukkala	2014	1	0	1961-2005	Norway	Norway	[36]
Pukkala	2014	1	0	1961-2005	Sweden	Sweden	[36]
Ide	2014	1	0	1984-2005	Scotland	UK	[37]
Paget-Bailly	2013	1	0	2001-2007	Bas-Rhin	France	[38]
Paget-Bailly	2013	1	0	2001-2007	Calvados	France	[38]
Paget-Bailly	2013	1	0	2001-2007	Doubs	France	[38]
Paget-Bailly	2013	1	0	2001-2007	Haut-Rhin	France	[38]
Paget-Bailly	2013	1	0	2001-2007	Hérault	France	[38]
Paget-Bailly	2013	1	0	2001-2007	Isére	France	[38]
Paget-Bailly	2013	1	0	2001-2007	Loire-Atlantique	France	[38]
Paget-Bailly	2013	1	0	2001-2007	Manche	France	[38]
Paget-Bailly	2013	1	0	2001-2007	Somme	France	[38]
Paget-Bailly	2013	1	0	2001-2007	Vendée	France	[38]
Demers	2011	1	0	1960	Denmark	Denmark	[39]
Demers	2011	1	0	1960	Finland	Finland	[39]
Demers	2011	1	0	1960	Iceland	Iceland	[39]
Demers	2011	1	0	1960	Norway	Norway	[39]
Demers	2011	1	0	1960	Sweden	Sweden	[39]
Demers	2011	1	0	1970	Denmark	Denmark	[39]
Demers	2011	1	0	1970	Finland	Finland	[39]
Demers	2011	1	0	1970	Iceland	Iceland	[39]
Demers	2011	1	0	1970	Norway	Norway	[39]

Demers	2011	1	0	1970	Sweden	Sweden	[39]
Demers	2011	1	0	1980	Denmark	Denmark	[39]
Demers	2011	1	0	1980	Finland	Finland	[39]
Demers	2011	1	0	1980	Iceland	Iceland	[39]
Demers	2011	1	0	1980	Norway	Norway	[39]
Demers	2011	1	0	1980	Sweden	Sweden	[39]
Demers	2011	1	0	1990	Denmark	Denmark	[39]
Demers	2011	1	0	1990	Finland	Finland	[39]
Demers	2011	1	0	1990	Iceland	Iceland	[39]
Demers	2011	1	0	1990	Norway	Norway	[39]
Demers	2011	1	0	1990	Sweden	Sweden	[39]
Gaertner	2004	1	0	1994-1997	Great Britain	UK	[40]

References

1. Roséństock, L.; Demers, P.; Heyer, N.J.; Barnhart, S. Respiratory mortality among firefighters. *Br. J. Ind. Med.* **1990**, *47*, 462-465, doi:10.1136/oem.47.7.462.
2. Heyer, N.; Weiss, N.S.; Demers, P.; Rosenstock, L. Cohort mortality study of seattle fire fighters: 1945-1983. *Am. J. Ind. Med.* **1990**, *17*, 493-504, doi:https://doi.org/10.1002/ajim.4700170407.
3. Lee, D.J.; Koru-Sengul, T.; Hernandez, M.N.; Caban-Martinez, A.J.; McClure, L.A.; Mackinnon, J.A.; Kobetz, E.N. Cancer risk among career male and female Florida firefighters: Evidence from the Florida Firefighter Cancer Registry (1981-2014). *Am. J. Ind. Med.* **2020**, *63*, 285-299, doi:https://doi.org/10.1002/ajim.23086.
4. Vena, J.E.; Fiedler, R.C. Mortality of a municipal-worker cohort: IV. Fire fighters. *Am. J. Ind. Med.* **1987**, *11*, 671-684, doi:10.1002/ajim.4700110608.
5. Beaumont, J.J.; Chu, G.S.; Jones, J.R.; Schenker, M.B.; Singleton, J.A.; Piantanida, L.G.; Reiterman, M. An epidemiologic study of cancer and other causes of mortality in San Francisco firefighters. *Am. J. Ind. Med.* **1991**, *19*, 357-372, doi:10.1002/ajim.4700190309.
6. Demers, P.A.; Heyer, N.J.; Rosenstock, L. Mortality among firefighters from three northwestern United States cities. *Br. J. Ind. Med.* **1992**, *49*, 664-670, doi:10.1136/oem.49.9.664.
7. Burnett, C.A.; Halperin, W.E.; Lalich, N.R.; Sestito, J.P. Mortality among fire fighters: A 27 state survey. *Am. J. Ind. Med.* **1994**, *26*, 831-833, doi:https://doi.org/10.1002/ajim.4700260612.
8. Figgs, L.W.; Dosemeci, M.; Blair, A. United States non-Hodgkin's lymphoma surveillance by occupation 1984-1989: a twenty-four state death certificate study. *Am. J. Ind. Med.* **1995**, *27*, 817-835, doi:10.1002/ajim.4700270606.
9. Wende, K.E. A study of mortality among city of Buffalo fire fighters. Ph.D., State University of New York at Buffalo, United States -- New York, 1996.
10. Ma, F.; Lee, D.J.; Fleming, L.E.; Dosemeci, M. Race-specific cancer mortality in US firefighters: 1984-1993. *J. Occup. Environ. Med.* **1998**, *40*, 1134-1138, doi:10.1097/00043764-199812000-00014.
11. Krstev, S.; Baris, D.; Stewart, P.; Dosemeci, M.; Swanson, G.M.; Greenberg, R.S.; Schoenberg, J.B.; Schwartz, A.G.; Liff, J.M.; Hayes, R.B. Occupational risk factors and prostate cancer in U.S. blacks and whites. *Am. J. Ind. Med.* **1998**, *34*, 421-430, doi:10.1002/(sici)1097-0274(199811)34:5<421::aid-ajim2>3.0.co;2-t.
12. Baris, D.; Garrity, T.J.; Telles, J.L.; Heineman, E.F.; Olshan, A.; Zahm, S.H. Cohort mortality study of Philadelphia firefighters. *Am. J. Ind. Med.* **2001**, *39*, 463-476, doi:https://doi.org/10.1002/ajim.1040.
13. Krishnan, G.; Felini, M.; Carozza, S.E.; Miike, R.; Chew, T.; Wrensch, M. Occupation and adult gliomas in the San Francisco Bay Area. *J. Occup. Environ. Med.* **2003**, *45*, 639-647, doi:10.1097/01.jom.0000069245.06498.48.
14. Ma, F.; Fleming, L.E.; Lee, D.J.; Trapido, E.; Gerace, T.A.; Lai, H.; Lai, S. Mortality in Florida professional firefighters, 1972 to 1999. *Am. J. Ind. Med.* **2005**, *47*, 509-517, doi:10.1002/ajim.20160.
15. Ma, F.; Fleming, L.E.; Lee, D.J.; Trapido, E.; Gerace, T.A. Cancer incidence in Florida professional firefighters, 1981 to 1999. *J. Occup. Environ. Med.* **2006**, *48*, 883-888, doi:10.1097/01.jom.0000235862.12518.04.
16. Bates, M.N. Registry-based case–control study of cancer in California firefighters. *Am. J. Ind. Med.* **2007**, *50*, 339-344, doi:https://doi.org/10.1002/ajim.20446.
17. Greene, K.; Konety, B.; Stoller, M. Results from the San Francisco Firefighters bladder cancer screening study. *Journal of Urology* **2008**, *179*, 323-323, doi:10.1016/S0022-5347(08)60945-3.
18. Zeig-Owens, R.; Webber, M.P.; Hall, C.B.; Schwartz, T.; Jaber, N.; Weakley, J.; Rohan, T.E.; Cohen, H.W.; Derman, O.; Aldrich, T.K.; et al. Early assessment of cancer outcomes in New York City firefighters after the 9/11 attacks: an observational cohort study. *Lancet (London, England)* **2011**, *378*, 898-905, doi:10.1016/S0140-6736(11)60989-6.
19. Zeig-Owens, R. Diagnostic Procedures Using Radiation and Risk of Thyroid Cancer: Causal Association or Detection Bias? An Examination of Population Cancer Trends and Data from the NYC Fire Department. City University of New York, 2015.
20. Karami, S.; Colt, J.S.; Schwartz, K.; Davis, F.G.; Ruterbusch, J.J.; Munuo, S.S.; Wacholder, S.; Stewart, P.A.; Graubard, B.I.; Rothman, N.; et al. A case–control study of occupation/industry and renal cell carcinoma risk. *BMC Cancer* **2012**, *12*, 344, doi:10.1186/1471-2407-12-344.
21. Daniels, R.D.; Kubale, T.L.; Yiin, J.H.; Dahm, M.M.; Hales, T.R.; Baris, D.; Zahm, S.H.; Beaumont, J.J.; Waters, K.M.; Pinkerton, L.E. Mortality and cancer incidence in a pooled cohort of US firefighters from San Francisco, Chicago and Philadelphia (1950–2009). *Occup. Environ. Med.* **2014**, *71*, 388-397, doi:10.1136/oemed-2013-101662.

22. Daniels, R.D.; Bertke, S.; Dahm, M.M.; Yiin, J.H.; Kubale, T.L.; Hales, T.R.; Baris, D.; Zahm, S.H.; Beaumont, J.J.; Waters, K.M.; et al. Exposure–response relationships for select cancer and non-cancer health outcomes in a cohort of US firefighters from San Francisco, Chicago and Philadelphia (1950–2009). *Occup. Environ. Med.* **2015**, *72*, 699-706, doi:10.1136/oemed-2014-102671.
23. Tsai, R.J.; Luckhaupt, S.E.; Schumacher, P.; Cress, R.D.; Deapen, D.M.; Calvert, G.M. Risk of cancer among firefighters in California, 1988–2007. *Am. J. Ind. Med.* **2015**, *58*, 715-729, doi:https://doi.org/10.1002/ajim.22466.
24. Moir, W.; Zeig-Owens, R.; Daniels, R.D.; Hall, C.B.; Webber, M.P.; Jaber, N.; Yiin, J.H.; Schwartz, T.; Liu, X.; Vossbrinck, M.; et al. Post-9/11 cancer incidence in World Trade Center-exposed New York City firefighters as compared to a pooled cohort of firefighters from San Francisco, Chicago and Philadelphia (9/11/2001-2009). *Am. J. Ind. Med.* **2016**, *59*, 722-730, doi:10.1002/ajim.22635.
25. Muegge, C.M.; Zollinger, T.W.; Song, Y.; Wessel, J.; Monahan, P.O.; Moffatt, S.M. Excess mortality among Indiana firefighters, 1985-2013. *Am. J. Ind. Med.* **2018**, *61*, 961-967, doi:10.1002/ajim.22918.
26. Landgren, O.; Zeig-Owens, R.; Giricz, O.; Goldfarb, D.; Murata, K.; Thoren, K.; Ramanathan, L.; Hultcrantz, M.; Dogan, A.; Nwankwo, G.; et al. Multiple Myeloma and Its Precursor Disease Among Firefighters Exposed to the World Trade Center Disaster. *JAMA Oncology* **2018**, *4*, 821-827, doi:10.1001/jamaoncol.2018.0509.
27. Lenahan, P. 30-year study of cancer incidence in firefighters and police officers in New Jersey's four largest municipalities. Rutgers University, 2018.
28. Hansen, E.S. A cohort study on the mortality of firefighters. *Br. J. Ind. Med.* **1990**, *47*, 805-809, doi:10.1136/oem.47.12.805.
29. Ide, C.W. Failing firefighters: A survey of causes of death and ill-health retirement in serving firefighters in Strathclyde, Scotland from 1985–94. *Occupational Medicine* **1998**, *48*, 381-388, doi:10.1093/occmed/48.6.381.
30. Deschamps, S.; Momas, I.; Festy, B. Mortality Amongst Paris Fire-Fighters. *European Journal of Epidemiology* **1995**, *11*, 643-646.
31. Tornling, G.r.; Gustavsson, P.; Hogstedt, C. Mortality and cancer incidence in stockholm fire fighters. *Am. J. Ind. Med.* **1994**, *25*, 219-228, doi:https://doi.org/10.1002/ajim.4700250208.
32. Kullberg, C.; Andersson, T.; Gustavsson, P.; Selander, J.; Tornling, G.; Gustavsson, A.; Bigert, C. Cancer incidence in Stockholm firefighters 1958–2012: an updated cohort study. *Int. Arch. Occup. Environ. Health* **2018**, *91*, 285-291, doi:10.1007/s00420-017-1276-1.
33. Petersen, K.K.U.; Pedersen, J.E.; Bonde, J.P.; Ebbehoej, N.E.; Hansen, J. Long-term follow-up for cancer incidence in a cohort of Danish firefighters. *Occup. Environ. Med.* **2018**, *75*, 263-269, doi:10.1136/oemed-2017-104660.
34. Bigert, C.; Gustavsson, P.; Straif, K.; Taeger, D.; Pesch, B.; Kendzia, B.; Schüz, J.; Stücker, I.; Guida, F.; Brüske, I.; et al. Lung cancer among firefighters: smoking-adjusted risk estimates in a pooled analysis of case-control studies. *J. Occup. Environ. Med.* **2016**, *58*, 1137-1143, doi:10.1097/JOM.0000000000000878.
35. Amadeo, B.; Marchand, J.-L.; Moisan, F.; Donnadieu, S.; Gaëlle, C.; Simone, M.-P.; Lembeye, C.; Imbernon, E.; Brochard, P. French firefighter mortality: analysis over a 30-year period. *Am. J. Ind. Med.* **2015**, *58*, 437-443, doi:10.1002/ajim.22434.
36. Pukkala, E.; Martinsen, J.I.; Weiderpass, E.; Kjaerheim, K.; Lynge, E.; Tryggvadottir, L.; Sparén, P.; Demers, P.A. Cancer incidence among firefighters: 45 years of follow-up in five Nordic countries. *Occup. Environ. Med.* **2014**, *71*, 398-404, doi:10.1136/oemed-2013-101803.
37. Ide, C.W. Cancer incidence and mortality in serving whole-time Scottish firefighters 1984–2005. *Occupational Medicine* **2014**, *64*, 421-427, doi:https://doi.org/10.1093/occmed/kqu080.
38. Paget-Bailly, S.; Guida, F.; Carton, M.; Menvielle, G.; Radoï, L.; Cyr, D.; Schmaus, A.; Cénée, S.; Papadopoulos, A.; Févotte, J.; et al. Occupation and head and neck cancer risk in men: results from the ICARE study, a French population-based case-control study. *J. Occup. Environ. Med.* **2013**, *55*, 1065-1073, doi:10.1097/JOM.0b013e318298fae4.
39. Demers, P.; Martinsen, J.I.; Weiderpass, E.; Kjærheim, K.; Lynge, E.; Sparén, P.; Pukkala, E. Cancer incidence among Nordic firefighters. *Occup. Environ. Med.* **2011**, *68*, A19-A20, doi:10.1136/oemed-2011-100382.60.
40. Gaertner, R.R.W.; Trpeski, L.; Johnson, K.C.; Canadian Cancer Registries Epidemiology Research, G. A case-control study of occupational risk factors for bladder cancer in Canada. *Cancer causes & control: CCC* **2004**, *15*, 1007-1019, doi:10.1007/s10552-004-1448-7.
